# Pentalogy of Cantrell in Two Neonate Littermate Puppies: A Spontaneous Animal Model Suggesting Familial Inheritance

**DOI:** 10.3390/ani13132091

**Published:** 2023-06-24

**Authors:** Wilson So, Shannon L. Donahoe, Juan M. Podadera, Hamutal Mazrier

**Affiliations:** Sydney School of Veterinary Science, Faculty of Science, The University of Sydney, Sydney, NSW 2006, Australia; wilson.so@sydney.edu.au (W.S.); shannon.donahoe@sydney.edu.au (S.L.D.);

**Keywords:** canine, congenital, ectopia cordis, gastroschisis, midline closure defect, papillon, rare disease, thoracoabdominal syndrome

## Abstract

**Simple Summary:**

Two papillon puppies (a stillborn and a live pup) were born with severe syndromic developmental abnormalities, involving incomplete fusion of the chest and abdominal wall with extrusion of internal organs. A similarly affected stillborn pup from the dam’s previous litter was reported, suggesting a possible familial inheritance. This study describes the results of advanced imaging (by computed tomography) and postmortem examinations of the two affected puppies compared to an unaffected stillborn papillon puppy from a separate litter. The results confirmed a diagnosis of Pentalogy of Cantrell, a rare syndrome, which is proposed to be inherited in humans. There are infrequent reports of this congenital condition in domestic animals with variable expression of the typical five signs of this syndrome, without any identified cause or confirmed genetic familial basis.

**Abstract:**

Developmental anomalies are an important cause of stillbirth and early perinatal death in companion animals. Many of these disorders remain poorly understood and provide an opportunity as a spontaneous animal model for human disease. Pentalogy of Cantrell is a rare congenital syndrome described in human neonates. It is a ventral midline closure defect with a proposed familial inheritance in humans. This syndrome involves five defects, including the thoracoabdominal wall, sternal, diaphragmatic, pericardial and cardiac malformations. Diverse expressions of these defects have been described in humans and sporadically in domestic animals. This severe syndrome commonly harbors a poor prognosis, posing an ethical and surgical dilemma. To better understand this syndrome and its presentation in dogs, we describe two rare cases of Pentalogy of Cantrell in a litter of papillon dogs. The affected puppies had anomalies compatible with the Pentalogy of Cantrell, including thoracoabdominal schisis, ectopia cordis, sternal cleft, pericardial agenesis, and diaphragmatic defects. The diagnosis was confirmed by advanced imaging (computed tomography) and postmortem examinations. The family history of this litter was explored and other cases in domestic animals were reviewed. This is the first report of the complete Pentalogy of Cantrell with ectopia cordis in the dog and the only report on papillons. Similar to human cases, possible familial inheritance and suspected male gender bias were observed. Further research on this novel animal model, its pathogenesis and its hereditary basis, may be helpful in better understanding this rare developmental disorder.

## 1. Introduction

Congenital developmental anomalies in most mammals, including humans and domestic companion animals, are infrequent. Despite their recognition, many malformations remain incompletely understood. Consequences of these anomalies can range from negligible to lethal. The estimated occurrence of stillbirth in puppies is reported to range between 3.7 and 7.0% [1,2]. An Australian epidemiologic study [2] estimated that 1.7% of stillbirth and early neonatal mortality in puppies are caused by congenital abnormalities (*n* = 44 out of 2574 puppies). Given this, developmental anomalies in newborn companion animals provide an opportunity as spontaneous animal models for rare inherited human diseases and syndromes [3,4,5]. 

Midline closure defects are an important group of infrequently inherited developmental anomalies that result from a fault in the fusion of midline structures early in fetal development [6,7]. As a consequence, a separation/splitting of the midline body wall (‘*Schisis*’) retains. These defects include a spectrum of malformations from small (incomplete xiphoid) to large (*thoracoabdominal schisis*), with the latter commonly exposing internal viscera [8], and is thus lethal to the fetus’s/newborn’s survival and/or resulting in pregnancy loss. 

A severe syndrome of midline closure defects has been described in humans as the Pentalogy of Cantrell (POC) [9], and within some of the literature has been synonymously defined as a thoracoabdominal syndrome (TAS) [10]. The Pentalogy of Cantrell involves a constellation of five congenital defects, including the thoracoabdominal wall, sternal, diaphragmatic, pericardial and cardiac defects [9]. Variable expressions of this have been described within the human literature with widely applied stratified phenotypes, including complete (all five defects; class I), probable/incomplete (four defects, including cardiac and ventral wall defects; class II) and a milder incomplete form (any combination of the above defects with sternal defects; class III) [11,12]. Advances in neonatology and surgical expertise have allowed for the treatment of some human cases. However, the majority of cases continue to harbor significant challenges and notable morbidity and mortality [12]. 

Only a few sporadic cases of POC have been described in the veterinary literature (see the literature search section below). Given the rarity of this syndrome, the current case report aims to provide detailed descriptions of the advanced imaging (computed tomography, CT) and postmortem findings from two newborn puppies with symptoms compatible with POC. To consider a possible inheritance mode and familial basis, this report investigates the dam’s breeding history and analyzes the pedigree of the cases reported here. Finally, we review previous cases from the veterinary literature and discuss the current understanding of the pathogenesis of POC, aiming to explore a proposed inheritance of this rare ventral midline closure defect in domestic animals.

## 2. Materials and Methods

### 2.1. Case Presentation at Birth

**Figure 1:**
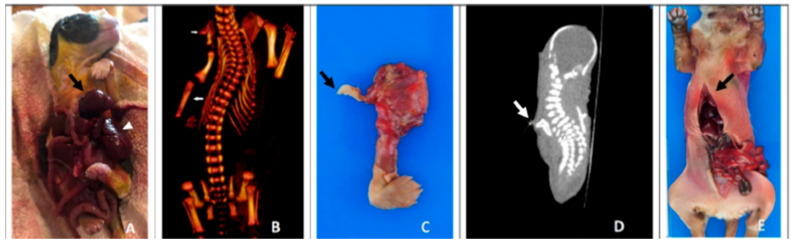
Presentation of midline defects in affected papillon puppies. (**A**) Case 1 with ectopia cordis (arrow) and extrusion of most abdominal viscera (arrowhead: liver). (**B**) Computed tomography (CT) of Case 1 highlighting lack of sternum, missing right radial bone (large arrow) and extranumerary right limb (small arrow). (**C**,**D**; ex situ and CT) Case 1 with an extranumerary limb (arrow[s]). (**E**) Case 2 with a thoracoabdominal defect; note the slightly fused cranial sternum compared to Case 1.

Two males in a litter of five full-term papillon puppies were born with variably severe ventral midline defects (thoracoabdominal schisis), including extrusion of the abdominal viscera. One was stillborn (Case 1; shown in Figure 1A), whilst the other (Case 2, shown in Figure 1E) was born alive and noted to be breathing. However, given the severe presentation of clinical signs, the live pup was humanely euthanized soon after birth (on the first day of life). The remaining three puppies of the litter (2 females and 1 male) were born alive without any identifiable gross congenital defects, according to the owner’s report.

### 2.2. Familial History

The dam (Dam 1) was approximately 2 years old at the birth of this litter and clinically healthy without any identified developmental anomalies or inherited defects. Approximately 6 months prior, the dam had its first litter of 4 puppies which included one stillborn puppy (Case 3; unknown gender; not examined in this study) and 3 live females. The stillborn in this previous litter had features of extensive thoracoabdominal malformation, similar to the two affected cases described in this report. Unfortunately, no specific distinguishing features were provided about Case 3. The breeder reported that both pregnancies were without any known pre-natal or peri-natal complications or illnesses. The two matings were with different healthy sires. The sire of the reported litter (Sire 1) was 3 years old and had fathered 3 litters previously. The sire of the previous litter (Sire 2) was approximately 1 year old and bred for the first time. Both sires have been mated since and each has successfully produced 3 additional litters, consisting of both live female and male offspring (Sire 1: 12 males, 15 females; Sire 2: 6 males, 5 females). No information was available regarding the incidence of stillborn puppies from the other litters from these sires, nor if similar defects were noted in any of their other offspring. The coefficient of inbreeding (COI) for Case 1 and Case 2 was 0.04%, whilst the previous litter (Case 3) had a COI of 5.60%. Hence, both litters had COIs that were not significant for inbreeding.

To compare gross and radiographic findings, an unaffected stillborn, full-term, female papillon puppy was used as a control. This female was born from a mating between the remaining unaffected male of this litter to a related dam. The COI for this control was 18.09%, with Sire1 being the grandfather of both maternal and paternal sides and Dam1 being the paternal grandmother. 

### 2.3. Postmortem Investigations

Frozen cadaver samples were submitted for further investigation, with the owner’s consent, and approved by the University of Sydney Animal Ethics Committee (AEC protocol 2018-1449). A veterinary embryologist (HM) performed an initial assessment of the cadavers for gross congenital anomalies. Whole-body computed tomography (CT) was conducted by a veterinary radiologist (JMP), using our previously published CT parameters [13]. Veterinary anatomical pathologists (WS, SLD) performed routine postmortem examination and histopathology. 

### 2.4. Literature Review of POC in Domestic Animals

Literature review was conducted to search for reports on either complete or incomplete POC in domestic animals, using the following databases: Web of Science Core collection (www.webofscience.com, accessed on 14 May 2023), Online Mendelian Inheritance in Animals (OMIA; www.omia.org, accessed on 14 May 2023) and Google Scholar (https://scholar.google.com, accessed on 14 May 2023). The search terms used were: ‘Pentalogy of Cantrell’ OR ‘Cantrell pentalogy’ OR ‘Thoracoabdominal syndrome’ OR ‘Thoracoabdominal schisis’ OR ‘gastroschisis’ OR ‘ectopia cordis’ OR ‘peritoneopericardial diaphragmatic hernia’ (aka ‘PPDH’) AND common domestic species names: ‘Cat’, ‘Dog’, ‘Goat’, ‘Cow’/’Bovine’, ‘Horse’, ‘Pig’/’piglet’/’Swine’, OR ‘domestic animals’. Additionally, we aimed to capture reports on cases with incomplete presentations of POC. These cases may have not been diagnosed as POC but reported a partial/incomplete presentation of some of the typical five clinical defects of POC. This was performed by manually screening the reference lists from the identified publications. 

## 3. Results

### 3.1. Imaging and Postmortem Findings

**Figure 2:**
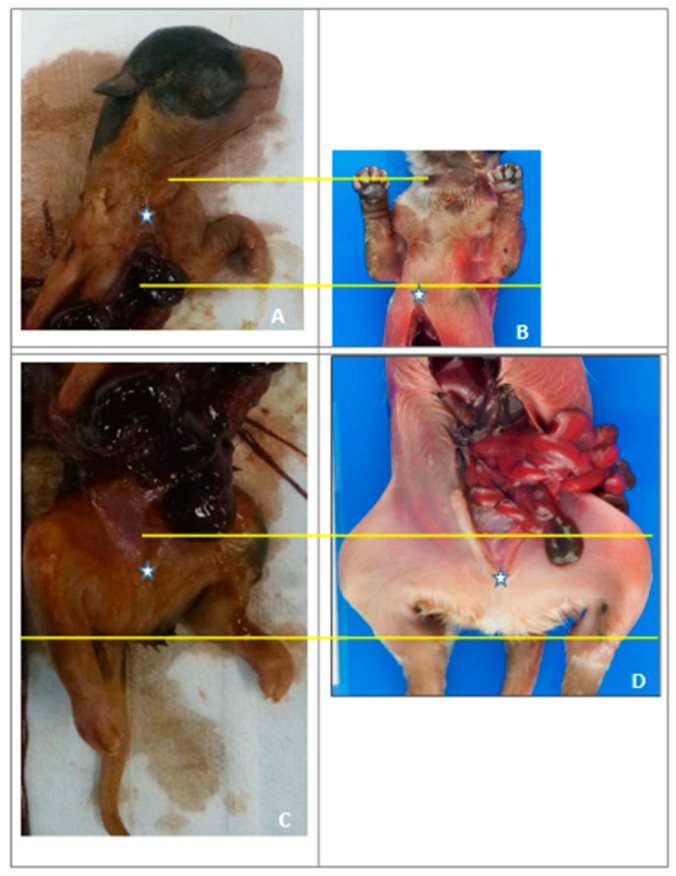
Cranial (thoracic) and caudal (abdominal) boundaries of midline schisis in affected papillon puppies. Left: Case 1 (**A**,**C**), right: Case 2 (**B**,**D**). Horizontal lines represent parallel anatomical markers (neck, elbow, knee and caudal midline levels). Stars represent the extent of the cranial (**A**,**B**) and caudal (**C**,**D**) midline defects.

Taking into account that this is a toy dog breed, both of the pups were considered full term, with crown-rump lengths of 145 mm and 160 mm (Case 1 and Case 2, respectively) [14]. Both affected pups had varying degrees of ventral midline closure defects, which were more severe in Case 1 compared to Case 2. Case 1 lacked a discernible sternum and pericardium (pericardial agenesis) and had partial diaphragmatic hypoplasia (approximately 70% absent) (shown in Figure 2). There was extrusion of the heart (ectopia cordis), liver, spleen, omentum and most of the gastrointestinal tract (gastroschisis) from the caudal aspect of the stomach to the colon, through the ventral midline defect. Additionally, Case 1 had an extranumerary limb (polymyelia), which had no grossly discernible bone originating from the right dorsal spine of the scapula (Figure 1C), confirmed by CT (shown in Figure 1B,D). Histopathology revealed the limb contained a central core of hyaline cartilage. Further in this pup, there was no appreciable right radius with a complete and intact right ulna detailed on the CT (shown in Figure 1B). Incidentally, this case was noted to be bilaterally abdominally cryptorchid. Representative histology of the tissues did not show any significant findings.

In comparison, Case 2 had a similar but somewhat less severe midline defect with extrusion of the liver, small intestine and cecum (gastroschisis), and partial diaphragmatic hypoplasia (approximately 20% absent) recognized by a small (4 × 3 mm) ventral defect (shown in Figure 2; similar to control). The sternum was present and fused to the first 8 ribs (shown in Figure 1E). The heart was surrounded by pericardium and contained within the thorax. Skeletal malformations were not identified in this pup. Both testes were located inguinally. The lungs were partially air inflated, confirming the pup was born alive. Representative histology of the tissues did not show any significant histologic changes beyond confirming partial pulmonary inflation.

Detailed examination of the internal heart was unfortunately not possible in either of the affected cases. This was due to the very small size (approximately 10 mm diameter), accelerated autolysis (contributed by the extrusion of visceral contents and contamination with meconium), and freeze–thaw artifact. Therefore, complete class I POC could not be fully confirmed, though it was highly suspected in Case 1, whilst Case 2 was consistent with partial or incomplete POC (class II or III). 

Necropsy and CT of the control stillborn puppy confirmed an intact abdominal and thoracic wall with the fusion of the sternum to the first 8 ribs. Inspection of the lungs confirmed that this puppy has not taken a breath and histologic examination revealed some amniotic fluid aspiration, indicative of fetal distress in utero. All of the organs, including the heart and the gastrointestinal tract from the esophagus to the rectum, were fully developed without any gross or histologic features of developmental abnormalities.

### 3.2. Literature Review of POC in Domestic Animals

Findings from publications that have reported either complete or incomplete POC in domestic animals are summarized (shown in Table 1). The total number of POC cases in domestic animals comprised 1 calf [15], 10 dogs [16,17,18,19,20], 2 cats [21,22] and 6 piglets [23]. Except for the stillborn piglets, all other reports are of live cases. Complete class I POC was reported in one cat [21], four dogs [17,18] and all of the described piglets (*n* = 6) [23]. Of the four previously published canine case reports that specified gender [16,18,19,20], the four dogs with POC were male (three German shepherds and one border terrier). Another report on cocker spaniels [17] specified 3 male and 3 female littermates, of which, 1 female with incomplete presentation had died at 4 days old. However, the gender of the apparently completely affected 3 cocker spaniel puppies was not specified. Together with our 2 cases, a total of 9 out of 12 known canine cases were males. No gender bias was observed in the other reported veterinary species [15,21,22,23]. Information regarding the familial history and siblings was only provided for the affected calf and piglets. The calf was born as a twin to another unaffected female [15], whilst the piglets were from separate litters and the only affected of their respective siblings [23].

## 4. Discussion

The combination of lesions in the two affected pups was consistent with the rare human neonatal syndrome of POC. Complete POC includes defects in the caudal sternum, anterior diaphragm, abdominal midline, pericardial agenesis and cardiac malformations [9]. A spectrum of clinical severity of this disease is recognized, and Toyama’s classification is widely adopted (described in the Introduction) [12]. Autolysis and freeze–thaw artifacts limited detailed intracardiac examination in these two affected cases and, hence, congenital intracardiac malformations could not be confirmed, nor completely excluded. According to Toyama’s classification, it is proposed that Case 1 was a complete (class I) expression of the syndrome, whilst Case 2 was more consistent with incomplete presentation (class II or III).

Embryologically, Cantrell (1958) proposed that POC likely results from two developmental anomalies: a fault of the septum transversum and its adjacent intraembryonic mesoderm, in addition to a failure of migration and fusion of the primordial sternum [9]. The former is associated with head-to-tail folding and is responsible for forming the anterior diaphragm, inferior pericardium and cardiac structures. The latter is related to the lateral folding of the fetus, which leads to the failure of fusion of the lateral abdominal wall [24]. This conceptual embryonic pathogenesis holds true in our current understanding of this disease.

In humans, the consequences of POC vary depending on the severity of the defects. According to the National Organization for Rare Disorders (NORD; https://rarediseases.org/, accessed on 27 May 2023), cases with complete POC (class I) that exhibit ectopia cordis at birth are considered the most severe presentation of POC syndrome. The prognosis is especially poor in cases of complete class I expression, with reported mortalities of up to 82% in surgical cases with ectopia cordis [25]. Epidemiologically, studies suggest POC occurs in approximately 1 per 65,000 to 200,000 live human births [24]. Unfortunately, information regarding the incidence of POC related to perinatal termination or stillbirth has not been explored, suggesting that the true incidence of POC may be higher.

Within the veterinary literature, POC has very rarely been reported, with confirmed cases in cats, dogs, cattle and pigs (Table 1). The sporadic canine cases of POC were reported in border terriers, cocker spaniels, and German shepherds [16,17,18,19,20], but not in papillons. A severe complete class I expression of POC with ectopia cordis, which was presented in Case 1, was only previously described in stillborn pigs [23]. The true incidence of POC in domestic animals is likely higher, given underreported stillbirths and common early euthanasia of neonates with gross anomalies. Further, inconsistencies regarding the terminology of midline malformations within the veterinary literature exist. This could be attributed to a lack of awareness of POC syndrome. An example of this includes the report on cocker spaniels [17], which described a myriad of lesions compatible with complete (class I) POC (*n* = 3), though it was not defined accordingly, whilst other reports discuss POC compatibility, but prefer the diagnosis of peritoneal pericardial diaphragmatic hernias (PPDH) and/or sternal cleft [18,20]. Consensus amongst veterinarians is needed to ensure consistent reporting and the authors recommend adaption of POC when the syndromic features are present.

Based on the veterinary literature, the prognosis of POC appears more favorable in dogs and cats compared to humans, with successful surgical management in most cases. However, most of the reported cases exhibited milder clinical presentations than Case 1 and Case 2, with only supraumbilical hernia visible externally, and have undergone surgical correction at young ages. These dogs and cats had smaller defects without gross evisceration. Except for one cocker spaniel [17], canine and feline cases that have not survived postnatally or were not amendable to surgical repair have not been reported within the reviewed literature. Hence, drawing a prognosis based on these reports will have an inherent bias, and is likely to be an overestimate. Stillbirth and early neonatal mortality are fairly frequently reported in papillon puppies (2.7% and 4.9%, respectively; out of 550 papillon puppies investigated) [1]. Therefore, it is interesting to explore whether more puppies within the breed could have succumbed to the lethal symptoms of POC.

The pathogenesis of human POC remains unclear, and a specific genetic mutation responsible for the formation of the syndrome has not yet been identified. Several familial cases have been described in humans (e.g., [26]), though many cases remain sporadic. Interestingly, the Dam (Dam1) had a similarly affected pup (Case 3) from a mating with a different sire, suggesting a possible familial inheritance of the syndrome in dogs. To the author’s best knowledge, all other sporadically reported animal cases either were of isolated individuals or confined within one litter [17]. Hence, the cases reported here are the first within consecutive litters suggesting a possible familial basis of canine POC.

In the POC of humans, there is a gender bias in males [26,27], with an approximate predominance of males to females between 1.35:1 to 2.7:1 [27,28]. Interestingly, 7 out of the 10 previously reported canine cases of POC (Table 1) were male as were our cases, suggesting a possible male bias in dogs. Similar observations on gender were not supported when we examined the sporadic cases in cats, pigs or calves. Further epidemiological studies are required, though given the rarity of the syndrome may be difficult to validate. 

This male bias could suggest the involvement of an X-linked mutation, which has also been proposed in some human reports [24,29]. The human X chromosome has been previously linked to POC in humans (Xq27 loci, currently re-mapped to Xq25-q26.1) [29], and to Goltz–Gorlin Syndrome (Xp11.23 loci) that rarely occurred concurrently in some cases of POC [30,31]. Goltz–Gorlin Syndrome is an X-linked human autosomal dominant disorder, caused by a mutation in the porcupine O-acyltransferase (*PORCN*) gene. This gene belongs to the Wnt signaling pathway, which interestingly involves the development of the sternum and thoracic body wall [32]. Goltz–Gorlin Syndrome causes cutaneous, dental, ophthalmic, neurologic and sexual anomalies predisposing to the formation of basal cell carcinomas [31]. Other sporadic reports of gene mutations have been reported in individual POC cases with duplication of aldehyde dehydrogenase 1 family member A2 (*ALDH1A2*), a gene located in human Chromosome 15 [33]. *ALDH1A2* has an important function in vitamin A metabolism, which plays a critical role in the development of the heart and diaphragm [33]. Candidate gene variants have not been investigated in any veterinary cases and should be explored in future studies. 

The more severely affected pup had an extranumerary limb (polymelia) extending from the right scapula and radial agenesis. Biologically, animals with one congenital defect have an increased incidence of more malformations. In humans, POC has been described concurrently with various anomalies distinct from midline defects, including musculoskeletal and visceral malformations [12]. Similarly, the cat with complete POC also had ectrodactyly and other visceral malformations including a split liver and bilobed gallbladder [20]. Based on the available veterinary literature, additional congenital malformations are likely sporadic without an association with POC development [20]. However, developmental anomalies in other body systems could develop at the same early embryonic stage, when the folding and migration defects causing POC occur [9]. This highlights the importance for veterinary investigators to thoroughly examine for other congenital disorders in animals affected by POC.

This case report has several limitations. Firstly, the incomplete access to breeding records and litter outcomes from the two separate sires limited further understanding of prevalence within the sire lines. Secondly, the stillborn pup from the dam’s previous litter (Case 3) was not available for examination and its gender was unknown. Thirdly, the autolysis and freeze–thaw condition of the cadavers limited a detailed examination of the heart. Therefore, intracardiac anomalies could not be confirmed, nor excluded, in these described cases. However, complete class I POC was highly suspected in Case 1, which had four of the five criteria, ectopia cordis and severe presentation, compared to the previously described cases in dogs. Considering that a genetic mutation has not been identified yet, there are no known genetic tests available in dogs that would be relevant for diagnosing these two individuals. Further, genomics and gene sequencing were not within the scope of this publication, though tissues have been retained for future investigations. This is specifically important, as a companion and other domestic animals have been revealed to be essential models for understanding pathogenesis and genetics in inherited developmental anomalies of dorsal midline closure defects [5].

## 5. Conclusions

To the best of the authors’ knowledge, this is the first report of possible familial POC affecting consecutive litters of the same dam, the only description of complete POC syndrome with ectopia cordis in the dog and the first report of POC in the papillon breed. A review of the literature on POC in domestic animals was conducted and we described our current understanding of this complex syndrome. Similar to the reported human cases, we observed a male predominance amongst the reported canine cases, which warrants investigation of a possible X-linked mutation. The presented cases pose a potential animal model for the inherited basis of POC in humans, with opportunities for prospective analysis and further investigations into pathogenesis and possible genetic mapping.

## Figures and Tables

**Table 1:** Reported literature on cases of Pentalogy of Cantrell (POC) in domestic animals.

Species and Breed	Gender ^(b)^ (No.)	Thoracoabdominal	Sternal	Diaphragmatic	Pericardial	Cardiac	Refs.
Bovine							
Simmental	F (1)	Yes, mild	No	Yes	No	Yes	[15]
Canine							
border terrier	M (1)	Yes	Yes	Yes	Yes	No	[16]
cocker spaniel	F (1)	Yes	No	No	No	NA ^(c)^	[17]
	M (3); F (2)	Yes	Yes	Yes	Yes	Yes (3); No (2)	[17]
German shepherd	M (1)	Yes	Yes	Yes	Yes	No	[18]
	M (1)	Yes, mild	Yes	Yes	Yes	No	[19]
	M (1)	NA ^(c)^	Yes	Yes	Yes	No	[20]
Feline							
British shorthair	M (1)	Yes	Yes	Yes	Yes	Yes	[21]
domestic shorthair	F (1)	Yes, mild	Yes	Yes	Yes	No	[22]
Swine							
crossbreed ^(a)^	M (3); F (3)	Yes	Yes	Yes	Yes	Yes	[23]
**Species and Breed**	**Live at birth? (No.)**	**Last reported survival age ^(d)^**		**Suggested POC class**			**Refs.**
Bovine							
Simmental	Yes (1)	Euthanized at 1 w		Incomplete, class III			[15]
Canine							
border terrier	Yes (1)	22 m		Incomplete, class III			[16]
cocker spaniel	Yes (1)	Died at 4 d		Incomplete, class III			[17]
	Yes (5)	Live at 1 y		Complete, class I (3); & incomplete, class III (2)			[17]
German shepherd	Yes (1)	5 m		Complete, class I			[18]
	Yes (1)	9 m		Incomplete, class III			[19]
	Yes (1)	53 m		Incomplete, class III ^(e)^			[20]
Feline							
British shorthair	Yes (1)	27 m		Complete, class I			[21]
domestic shorthair	Yes (1)	Adult (unknown age)		Incomplete, class III			[22]
Swine							
crossbreed ^(a)^	No (6)	Stillborn		Complete, class I			[23]

(a) Landrace = large white Pietrain crossbreed. (b) M = male, F = female. (c) NA = not available. (d) d = days, m = months, w = weeks, y = years. (e) Listed as an alternative differential diagnosis.

## Data Availability

The data that support the findings of this study are available from the corresponding author [HM] upon reasonable request.
